# Reduced Dependence of Crested Ibis on Winter-Flooded Rice Fields: Implications for Their Conservation

**DOI:** 10.1371/journal.pone.0098690

**Published:** 2014-05-29

**Authors:** Yiwen Sun, Andrew K. Skidmore, Tiejun Wang, Hein A. M. J. van Gils, Qi Wang, Baoping Qing, Changqing Ding

**Affiliations:** 1 Department of Natural Resources, Faculty of Geo-Information Science and Earth Observation, University of Twente, Enschede, The Netherlands; 2 Bio-resources Key Laboratory of Shaanxi Province, Hanzhong, Shaanxi Province, China; 3 College of Biological Science and Engineering, Shaanxi University of Technology, Hanzhong, Shaanxi Province, China; 4 Shaanxi Hanzhong Crested Ibis National Nature Reserve, Hanzhong, Shaanxi Province, China; 5 College of Nature Conservation, Beijing Forestry University, Beijing, China; University of Lleida, Spain

## Abstract

The Crested Ibis *Nipponia nippon* was once thought to be extinct in the wild until seven birds were discovered in a remote mountain village in China in 1981. Studies suggested that winter-flooded rice fields play an essential role in nest site selection by the Crested Ibis and hence in their survival. Considerable efforts were therefore made to conserve the winter-flooded rice fields, but these have caused conflicts between the agricultural and conservation communities. The population and geographical range of the wild Crested Ibis has expanded greatly since 1981, but there is no spatial information on the winter-flooded rice fields, nor on the current association of nest sites and winter-flooded rice fields. We mapped winter-flooded rice fields across the entire current range of Crested Ibis using innovative remote sensing and geographical information systems (GIS) techniques. The spatial relationships between the nest site clusters and winter-flooded rice fields were quantified using Ward's hierarchical clustering method and Ripley's K-function. We show that both have significantly clumped distribution patterns and that they are positively associated. However, the dependence of Crested Ibis on the winter-flooded rice fields varied significantly among the nest site clusters and has decreased over the years, indicating the absence of winter-flooded rice fields is not constraining their recovery and population expansion. We therefore recommend that efforts should be made to protect the existing winter-flooded rice fields and to restore the functionality of natural and semi-natural wetlands, to encourage both *in-situ* conservation and the re-introduction of the Crested Ibis. In addition, we recommend that caution should be exercised when interpreting the habitat requirements of species with a narrow distribution, particularly when that interpretation is based only on their current habitat.

## Introduction

Studying habitats and how species select their nest sites yields important information about animal behavioural ecology and the conservation management of endangered species [1]–[3]. Selecting a suitable nest site is critical for birds because it directly affects their reproductive success and fitness [4]. Among all the characteristics of nest sites that influence breeding success, the availability of adequate forage may be a primary force for establishing the breeding territory of many avian species [5], [6].

**Figure 1:**
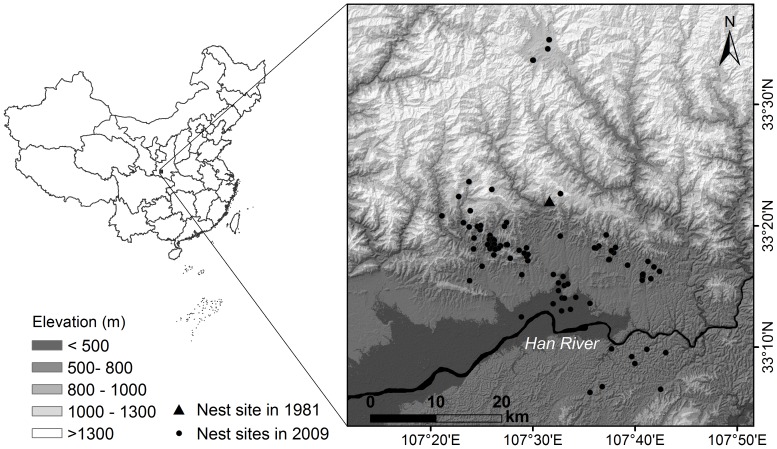
Map of the study area. The locations of nest sites of the Crested Ibis in 1981 and 2009 are marked.

The rice field forms one of the main artificial wetland ecosystems in China and sustains a diverse assemblage of plant and animal species [7]–[9]. In traditional agricultural systems, rice production involves single cropping and flooding the field while it lies fallow over a long period. The flooded rice fields provide a foraging habitat similar to natural and semi-natural wetlands for waterbirds during the winter and breeding season [10]–[12]. In terms of conservation value, the flooded rice fields contribute considerably more to waterbird biodiversity and success than unflooded fields [7]. Many birds benefit from this agricultural practice, including the endangered Crested Ibis *Nipponia nippon* [13]. The Crested Ibis was originally widespread in East Asia, but the population declined severely during the twentieth century [14]. This species was thought to be extinct in the wild until seven individuals were rediscovered in a small and remote village on the southern slopes of the Qinling Mountains in China in 1981 (Figure 1) [15]. Since 1981 this relict population has been carefully monitored and managed. The decline of the Crested Ibis was explained, in part of its distribution area, by the introduction of double cropping, i.e. replacing the winter flooding of rice fields by dryland farming of rapeseed or winter wheat, thereby reducing the foraging habitat during the winter and breeding seasons [14], [16].

**Figure 2:**
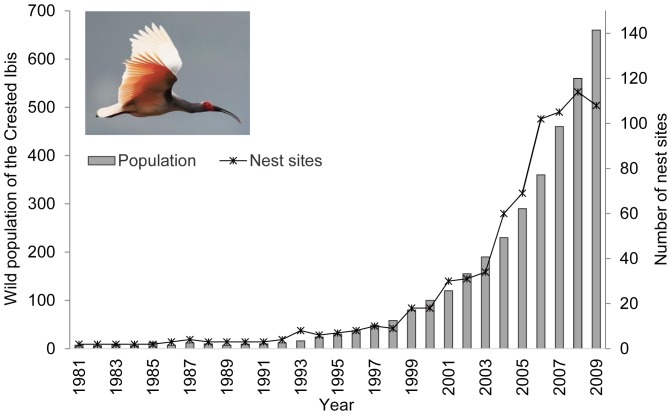
The size of the wild population of Crested Ibis and number of nest sites from 1981 to 2009.

Unfortunately, we know little about the historical breeding habitats of the Crested Ibis or why they selected their breeding locations, and most of the scientific research on this species has been carried out since their rediscovery. The small relict population in the Qinling Mountains had a distribution area of about 100 km^2^. A principal component analysis by Wang et al. [17] indicated one of their breeding habitat requirements was a short distance (74.1±42.1 m) to winter-flooded rice fields. Li et al. [18] used resource selection functions and found that the proximity of winter-flooded rice fields was an important factor in selecting their nest sites. A significant positive effect of winter-flooded rice fields on their selection of a nest site was confirmed by Ding [16]. As the wild population of the Crested Ibis continued to increase under protection, an aggregation of nest sites was observed, and these were reported to be associated with the distribution of winter-flooded rice fields [19]. In the early stage of the population recovery, almost all the researchers and conservationists interested in the Crested Ibis, as well as the local authorities and local community, thought that the presence of winter-flooded rice fields would determine the survival and recovery of the wild Crested Ibis. Hence, the conservation policy was focused on maintaining the traditional practice of winter-flooding of rice fields and compensating the farmers for their consequent loss of income [16]. Currently, the number of nest sites has increased from the original two to over 100 (Figure 2) and the distribution area of the Crested Ibis is 30 times larger, ranging from the mountains down to the Han River floodplain [20]. However, the winter-flooded rice fields are known to be very limited on the floodplain, based on the study of the local cropping system by Dong et al. [21]. This seemingly contradictory breeding behaviour of the Crested Ibis raises the question as to how the species has responded more recently to the distribution of winter-flooded rice fields, particularly because species-habitat relationships may vary over time and space [22]–[24].

There are no data or analyses on the distribution and extent of winter-flooded rice fields in the current distribution range of the wild Crested Ibis. Satellite-based remote sensing offers the opportunity to investigate the habitat availability for wildlife in time and space, and has been successfully applied to other bird ecology and conservation measures [11], [25], [26]. However, the temporal dynamic characteristics and the relatively narrow strips (which can be less than 30 m wide) of winter-flooded rice fields in the mountainous topography at Qinling pose challenges to traditional optical remote sensing methods. In the present study, we applied innovative remote sensing and geographical information systems (GIS) techniques to map winter-flooded rice fields, and used this information to explore the relationship between the distribution and extent of winter-flooded rice fields and the nest sites of the Crested Ibis over its entire range.

## Materials and Methods

### Study area and nest sites

The study area (3,980 km^2^) is located between 33°03′ to 33°39′N and 107°10′ to 107°54′E, at the intersection of the Han River plain and the southern slopes of the Qinling Mountains, China (Figure 1). The elevation ranges from 370 m to 2,400 m. The Crested Ibis is mainly seen in the plains and middle-lower mountain regions below 1,400 m. Major land covers in the study area include forest, shrub, grass, cropland, open water, built-up areas and waste land. The cropland can be divided into rain-fed fields with dryland crop cultivation, summer-irrigated rice fields with dry fallow or rotational dryland cultivation in winter (winter-dry rice fields), and rice fields with an intentionally or naturally flooded fallow period from November to May (winter-flooded rice fields). The Crested Ibis forages mainly in the winter-flooded rice fields, rivers and reservoirs for frogs, loaches, eels and winkles, both in winter and spring, prior to and during the breeding season, which starts in March and lasts to June [20].

A long-term demographic monitoring programme of the Crested Ibis (e.g. bird ringing and nest recording) has been carried out by the Shaanxi Crested Ibis Nature Reserve since 1981. Each year the study area is systematically searched on a weekly basis to locate nests from March to May. In the meantime, every public report of an ibis nest sites is also verified by experts from the nature reserve. The monitoring has expanded to new areas, including less accessible habitats, as the population has grown, and we therefore believe that all the nest sites have been identified during the census and monitoring. The geographical locations of the nest sites have been recorded using a global positioning system (GPS) since 2003, and the nest sites known before 2003 were revisited in 2004 to obtain GPS coordinates. The wild population of Crested Ibis has increased in the last three decades, particularly in the last decade (Figure 2). There were 102 nest sites in the study area in 2009, which we used to test our research questions (see Figure 1 for their spatial distribution).

### Classification of winter-flooded rice fields and associated major land cover/land use types

#### Preparation of satellite images and ancillary topographic data

Two cloud- and snow-free Landsat Thematic Mapper (TM) images (30 m resolution; 12 January 2009 and 5 June 2009) were selected to map the winter-flooded rice fields and associated major land cover/land use types. The TM images were geometrically rectified using 1∶50,000 topographic maps and the processing utilized a second-order polynomial and nearest neighbour interpolation, resulting in a root mean square error (RMSE) of less than 0.5 pixels (15 m). All image pre-processing was executed using ENVI 4.8 (Exelis Visual Information Solutions, Colorado, USA).

Topographic information, including elevation and terrain position, was derived from 30-m ASTER Global Digital Elevation Model (DEM) Version 2, released by METI and NASA on October 17, 2011, with vertical accuracies between 10 m and 25 m RMSE (https://lpdaac.usgs.gov/). The DEM data were geometrically co-registered with the TM images using a second-order polynomial and nearest neighbour interpolation. Five terrain position classes – gully, lower mid-slope, mid-slope, upper mid-slope, and ridge – were calculated using the algorithm [27] implemented in Interactive Data Language (IDL; Exelis Visual Information Solutions, Colorado, USA).

#### Land cover/land use field data collection

Permits and approvals were obtained from Shaanxi Hanzhong Crested Ibis National Nature Reserve for this study, including the fieldwork within the reserve. Fieldwork did not involve any endangered or protected species. The land cover/land use data for image classification and verification were collected in the field during April 2012, based on stratified random sampling. Seven strata (winter-flooded rice fields, winter-dry rice fields, rain-fed fields, open water, forest, shrub/grass, and other) were identified. The size of the sample plots was 50×50 m. The geographical coordinates of the plots' centres were recorded using a GPS receiver with a positional accuracy of 5–10 m. In total, we described 768 sample plots and randomly divided them into two equal groups for training and testing the classification.

#### Hybrid image classification approach

The satellite images were initially classified using a nonparametric supervised classification algorithm, called Support Vector Machine (SVM). The SVM algorithm locates the optimal hyperplane decision boundary that separates classes [28]. The classifier produced a land cover/land use map as well as seven rule maps in ENVI 4.8. Each rule map contained the probability that a pixel belonged to one of the seven classes.

A GIS-based expert system was constructed to perform post-classification sorting of the initial land cover/land use classification using additional topographic data (i.e. elevation and terrain position). The expert system was described in detail by Skidmore [29]. The land cover/land use map and the associated probability rule images were presented to the expert system as input. Elevation and terrain position data acted as items of evidence to infer the most probable land cover/land use type that would occur in a given grid cell. The key mechanism of the expert system is Bayes' theorem. Let *A* be an item of evidence known at a given location, and let us set up a hypothesis (*B_j_*) that the land cover/land use class *j* occurs at this location. The Bayes' rule can then be given as:
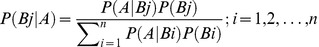
Equation(1)where *P*(*A*|*B_j_*) is the conditional probability that a certain grid cell location has an item of evidence (*A*) given *B_j_* (see Table S1); *P*(*B_j_*) is the *prior* probability of occurrence of a land cover/land use class at a certain location that can be obtained from the rule images of the initial classification. The denominator of Equation (1) is the sum of all possible cases where *n* is the total number of the land cover/land use classes in this study (i.e. *n* = 7).

The expert system algorithm worked forward from the item of evidence to the hypothesis and the search terminated only after all the evidence had been evaluated. All pixels were finally relabelled by the hypothesis that possessed the highest *posterior* probability (i.e. *P*(*B_j_*|*A*)). We expected the expert system would correct misclassifications generated by the SVM classifier. The expert system algorithm was programmed in C# with Microsoft NET framework.

**Figure 3:**
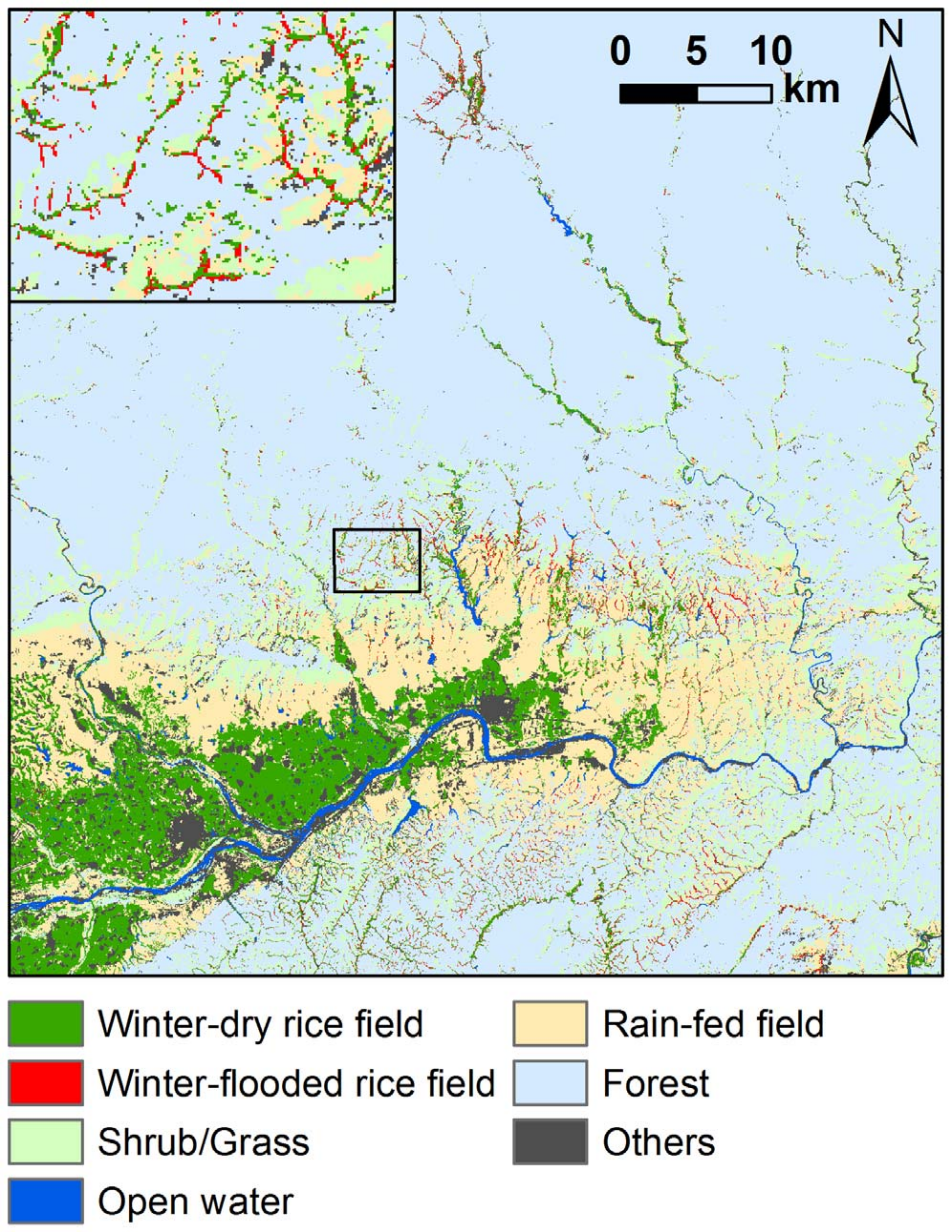
Land cover/land use map generated by the hybrid classifier. The inset shows a zoomed-in map of a specific sample area.

The accuracy of the land cover/land use map was assessed using a confusion matrix of the field observations (n = 384) against the classification result. The overall accuracy and Kappa statistic [30] were both improved by the expert system (see Table S2). The McNemar test for related samples [31], [32] confirmed the superiority of the hybrid classifier (χ^2^ = 34.03, *P*<0.001). The class of winter-flooded rice fields achieved a producer's and user's accuracy of 89.3% and 90.4%, respectively. The final map of all land cover/land use types is presented in Figure 3.

### Spatial statistical analyses

To investigate the spatial variation of winter-flooded rice field habitats, we quantified the abundance of such fields around nest sites. The average foraging distances of breeding Crested Ibis were observed to be at least 0.56±0.68 km and at most 2.82±1.69 km at different periods of the breeding season [33]. We therefore assumed a conservative radius of 3 km for buffers around the nest sites. The area of winter-flooded rice fields within the 3-km buffer around each nest was calculated using Spatial Analyst Tools in ArcGIS 10.0. The extent of the winter-flooded rice fields was distinguished using Ward's method [34], constrained *a priori* to three levels (i.e. high, medium and low) (see Figure S1). Ward's method is one of the most widely used hierarchical cluster analysis algorithms, and it has the advantage that it can select the optimal number of clusters and create clusters with a high degree of uniformity [35], [36]. Ward's method was also used to identify nest site clusters based on their geographical locations. The differences in the area of winter-flooded rice fields within the 3-km buffers around the nests were compared among the nest site clusters using the Kruskal-Wallis test followed by a pairwise Mann-Whitney U test.

Ripley's K-function [37] was used to further examine the spatial patterns of nest sites and winter-flooded rice fields, and to test whether the spatial distribution of the fields correlated with nest locations for all the sites together, as well as for each nest site cluster. The K-function is a tool for quantification of spatial pattern intensity of completely mapped point data (i.e. locations of all the nest sites or winter-flooded rice fields in a predefined study area), by utilizing a random species distribution over a range of distances [37], [38]. It compares the number of points within any distance to an expected number for a spatially random distribution. The classification data of the winter-flooded rice fields were converted from raster data to point features using the Conversion Tool in ArcGIS 10.0 (ESRI, Inc., Redlands, California, USA). We used the L-function, the linearized form of the K-function, to aid in interpretation and to stabilize the variance [38], [39]. Under the null model of complete spatial randomness (CSR), the theoretical value of the L-function is 0, whereas a positive value indicates a clumped distribution and a negative value indicates a regular distribution at a specific distance. The edge correction was superfluous because no nests were located outside the study area. The bivariate and multivariate generalizations of the K-function and L-function characterize the spatial associations between two or more variables [38], . The population independence hypothesis assumes that the location of points of a given population (i.e. nest sites or winter-flooded rice fields) is independent of the location of points of the other [41]. We therefore tested the bivariate L-function against the null hypothesis of population independence to determine the spatial relationship between the nest sites and winter-flooded rice fields. Theoretically, the L-function is positive if the two populations of points are positively associated and negative if they are negatively associated.

We used Monte Carlo simulations to generate 95% confidence envelopes for the L-function under the null hypothesis from 1,000 random permutations. If the observed L-function lies above the upper envelope (or below the lower envelope), the null hypothesis can be rejected. For the univariate L-function, positive differences between the observed L-function and the upper envelope of the null hypothesis of CSR indicate that the points are more clumped than expected by chance. Similarly, negative differences between the observed L-function and the lower envelope of the null hypothesis of CSR indicate that the points are more regular than expected by chance. For the bivariate L-function, positive differences between the observed L-function and the upper envelope of the null hypothesis of population independence indicate a spatial attraction between the two populations of points. Similarly, negative differences between the observed L-function and the lower envelope of the null hypothesis of population independence indicate a spatial repulsion between the two populations of points.

The univariate K-function and L-function were computed at 500 m intervals and using distances of 500 m to 30,000 m (i.e. half of the smallest edge length of the study area was taken as the largest distance for analysis set by the software package). The bivariate functions were calculated at 500 m intervals using distances of 500 m to 3,000 m based on the foraging distance range. All the calculations and simulations were computed using the R statistical package ‘ads’ (spatial point patterns analysis) [42]. In addition, the estimated L-functions were compared using Analysis of Variance (ANOVA) to confirm the differences in the spatial associations between nest sites and winter-flooded rice fields among the nest site clusters. Post-hoc pairwise comparisons were performed using Tukey's Honestly Significant Difference (HSD) test.

## Results

### Spatial patterns and associations of nest sites and winter-flooded rice fields

**Figure 4:**
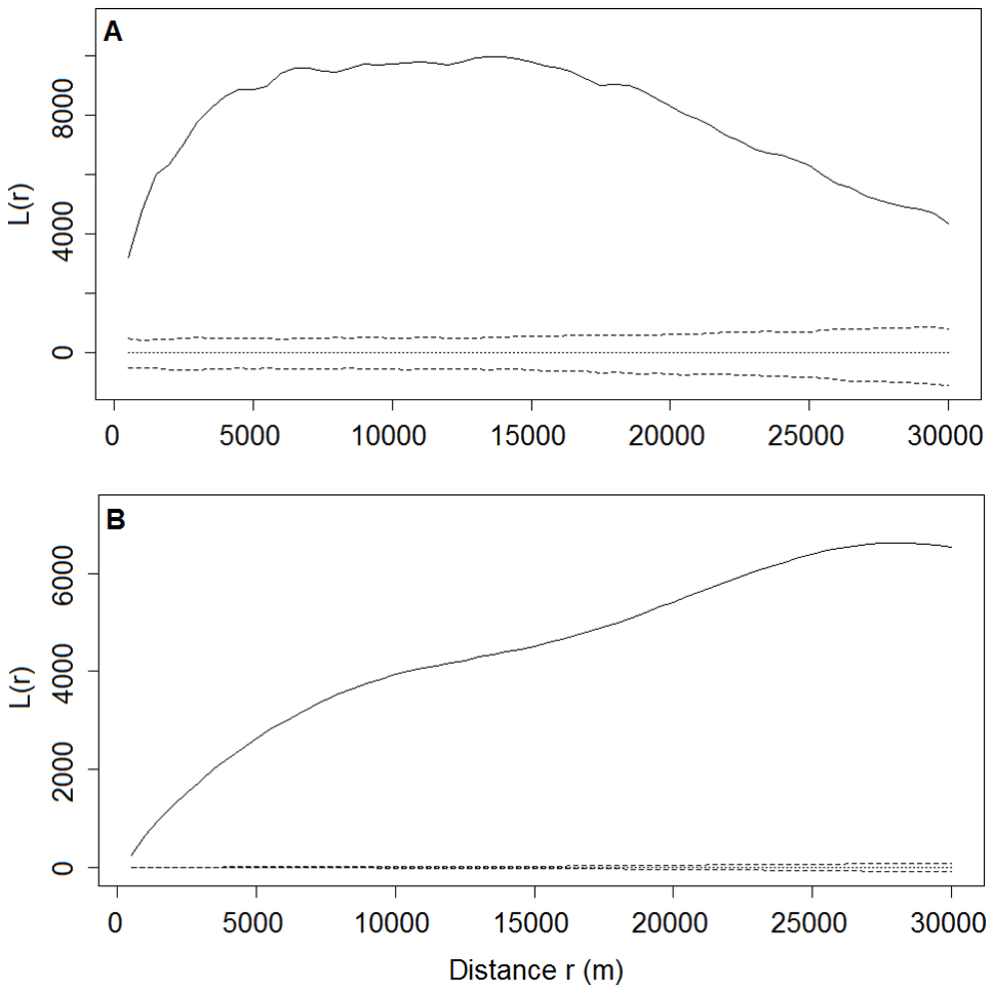
Plot of univariate L-function against distance r showing the spatial pattern. A. Distribution of nest sites. B. Distribution of winter-flooded rice fields. Solid lines are observed values of L(r). Dotted and dashed lines are the theoretical values and the 95% confidence envelopes, respectively, for the pattern expected from a random distribution. Values of L(r) above the upper envelope indicate a significantly clumped distribution at distance r. Values of L(r) below the lower envelope indicate a significantly regular distribution at distance r.

**Figure 5:**
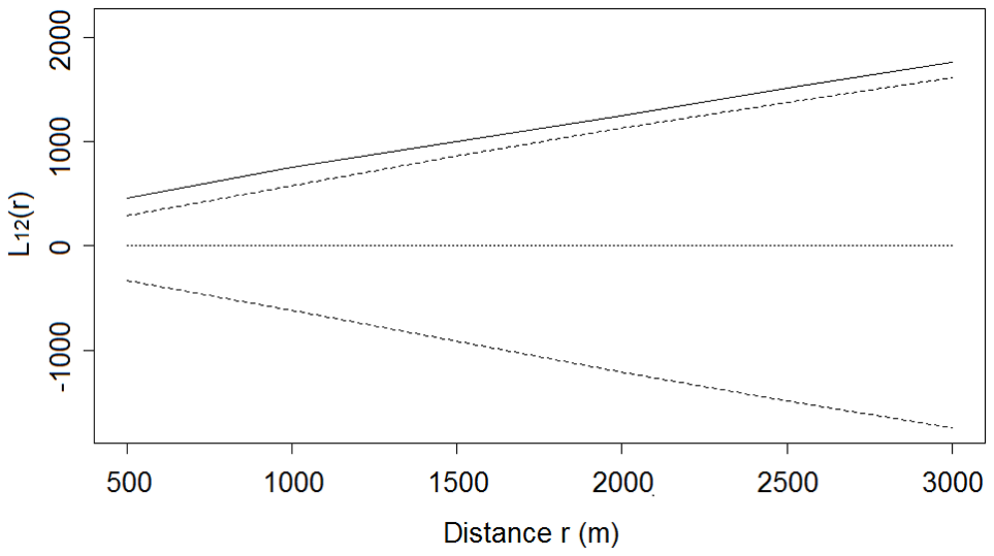
Plot of bivariate L-function for the spatial association between nest sites and winter-flooded rice fields. Solid lines are observed values of L_12_(r). Dotted and dashed lines are the theoretical values and the 95% confidence envelopes, respectively, for the pattern expected under the null hypothesis of population independence. Values of L_12_(r) above the upper envelope indicate a significant positive association, whereas values of L_12_(r) below the lower envelope indicate a significant negative association.

The values of the L-functions for the distributions of both nest sites and winter-flooded rice fields differed from zero and were above the upper bound of the 95% confidence interval at all distances from 500 m to 30,000 m (Figure 4), which led us to reject the null hypothesis of random distribution. These results indicate that the nest sites and winter-flooded rice fields were clumped, irrespective of distance. We also rejected the null hypothesis of population independence based on the bivariate L-function analysis, as the observed results lay outside the confidence envelope. A significant positive spatial association between the nest sites and winter-flooded rice fields was apparent at all distances from 500 m to 30,000 m (Figure 5).

### Nest site clusters and their association with winter-flooded rice fields

**Figure 6:**
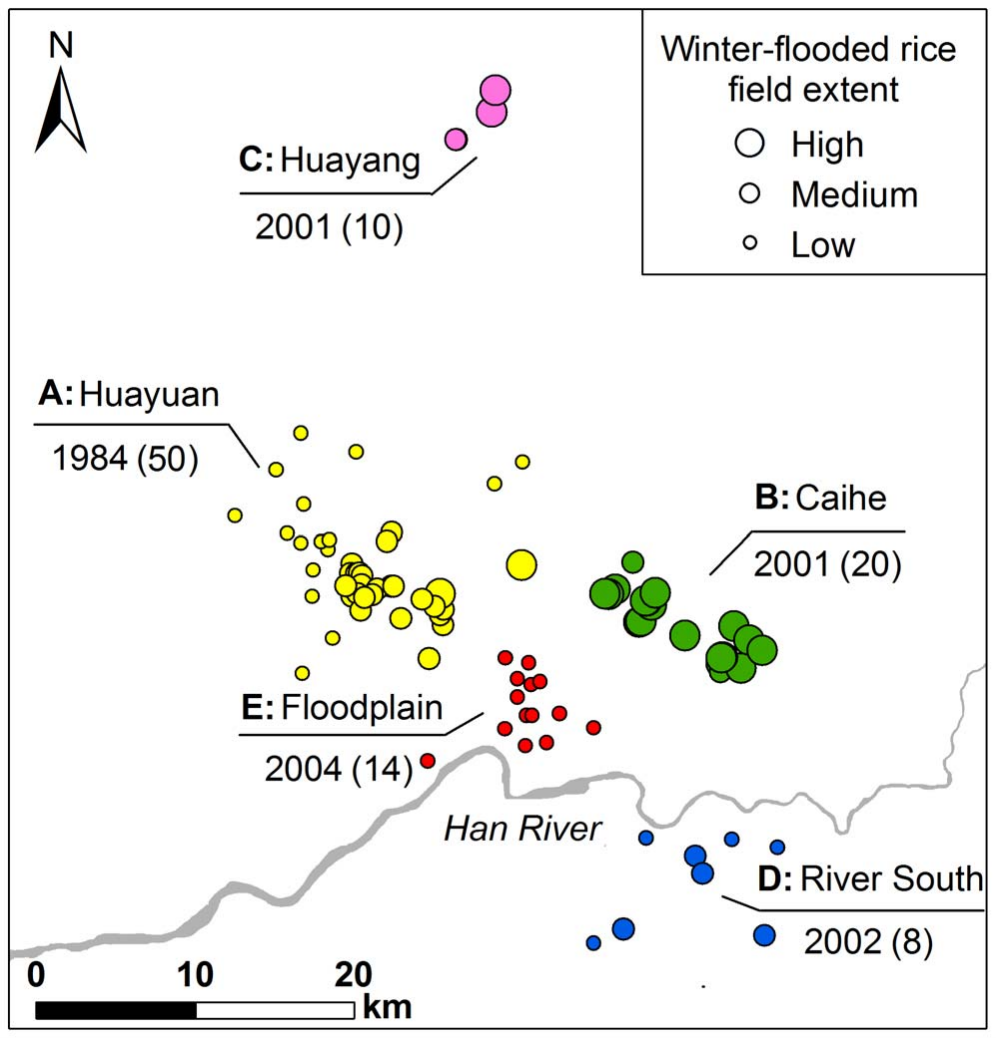
Nest site clusters and the extent of winter-flooded rice fields. Five clusters (A–E) were identified based on their geographical locations, using Ward's hierarchical clustering method. They are shown in different colours. The extent of winter-flooded rice fields within 3-km buffers around the nest sites are delineated by circles of three different sizes (high, medium and low). The locality name and year when the first nest site was established in each cluster are given above and below the line, with the total number of nests per cluster in parentheses.

**Figure 7:**
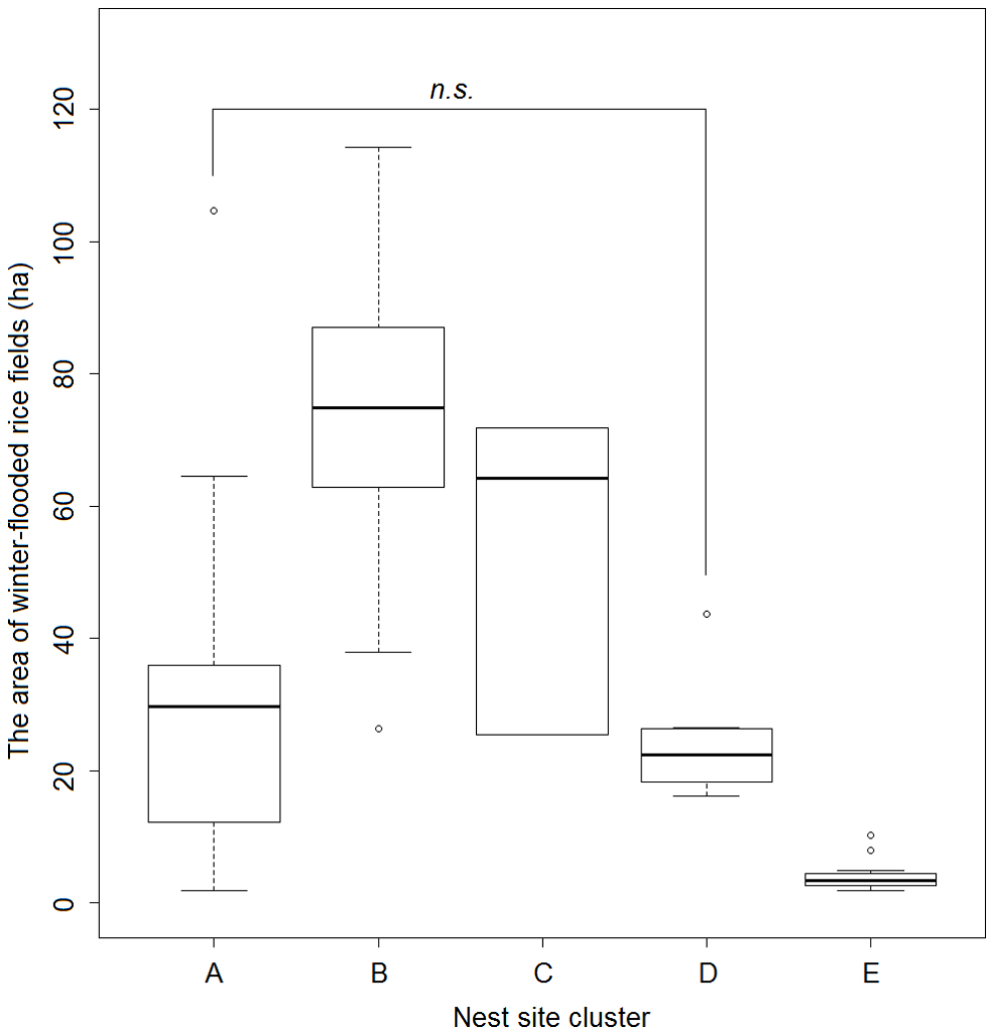
Differences in the area of winter-flooded rice fields in the nest site clusters. The area of winter-flooded rice fields within 3-km buffers around the nest sites in the five clusters (A–E) is shown by box plots. The only pair with no significant difference is annotated based on pairwise Mann-Whitney U tests (*P*≥0.05).

We distinguished five clusters of nest sites: Clusters A, B and C were on the southern slopes of the Qinling Mountains, Clusters D was to the south of the Han River, and Cluster E was on the floodplain. The extent of winter-flooded rice fields around the nest sites varied significantly among these five clusters (Kruskal-Wallis χ^2^ = 59.05, *P*<0.001) (Figures 6 and 7). The nests of Cluster E extended over the smallest area of the winter-flooded rice fields (4.13±2.29 ha), while Clusters B and C had a much greater extent (70.07±20.34 ha and 51.79±23.06 ha, respectively). Clusters A and D covered an intermediate area size (26.95±17.87 ha and 24.24±8.77 ha, respectively) and were not significantly different from each other (Figure 7).

**Figure 8:**
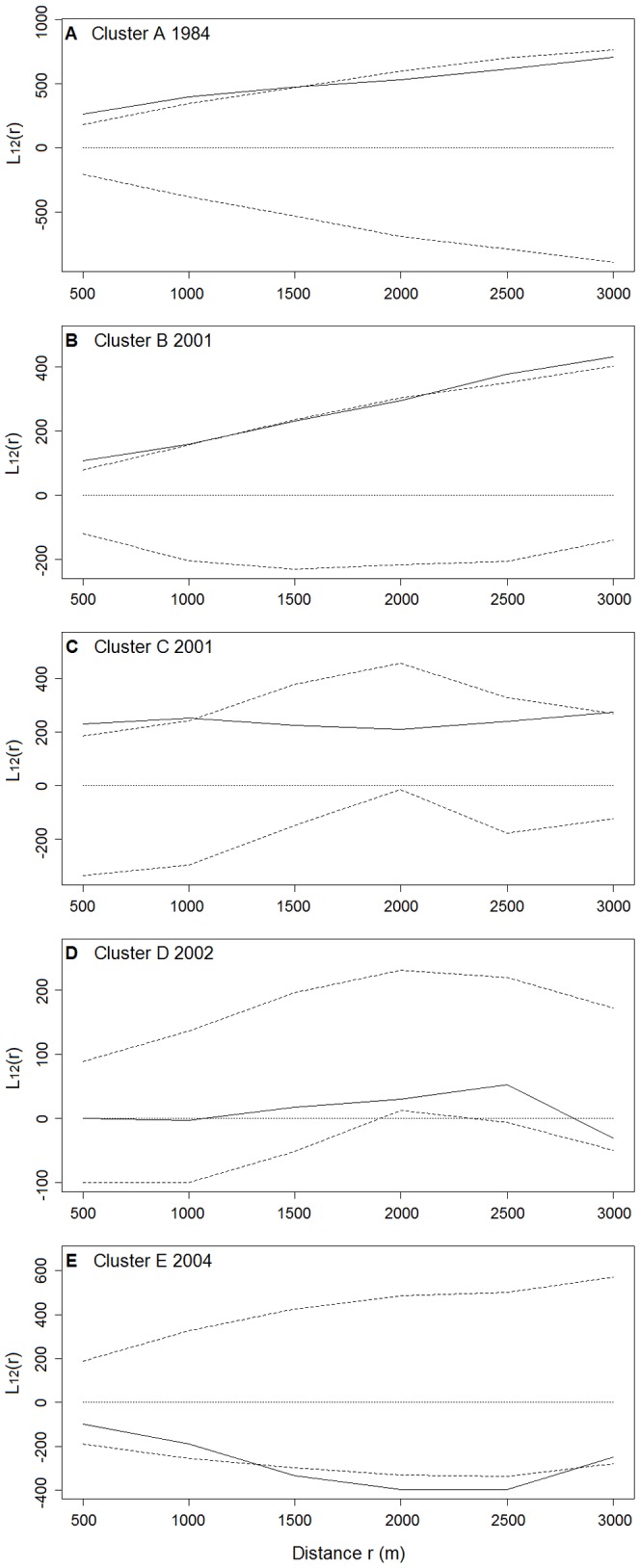
Spatial association between nest sites and winter-flooded rice fields in the nest site clusters. Bivariate L-function L_12_(r) against distance r for each cluster is plotted (A–E). Solid lines are observed values of L_12_(r). Dotted and dashed lines are the theoretical values and the 95% confidence envelopes, respectively, for the pattern expected under the null hypothesis of population independence. Values of L_12_(r) above the upper envelope indicate a significant positive association, whereas values of L_12_(r) below the lower envelope indicate a significant negative association. The year when the nest site was first established in the cluster is given.

**Figure 9:**
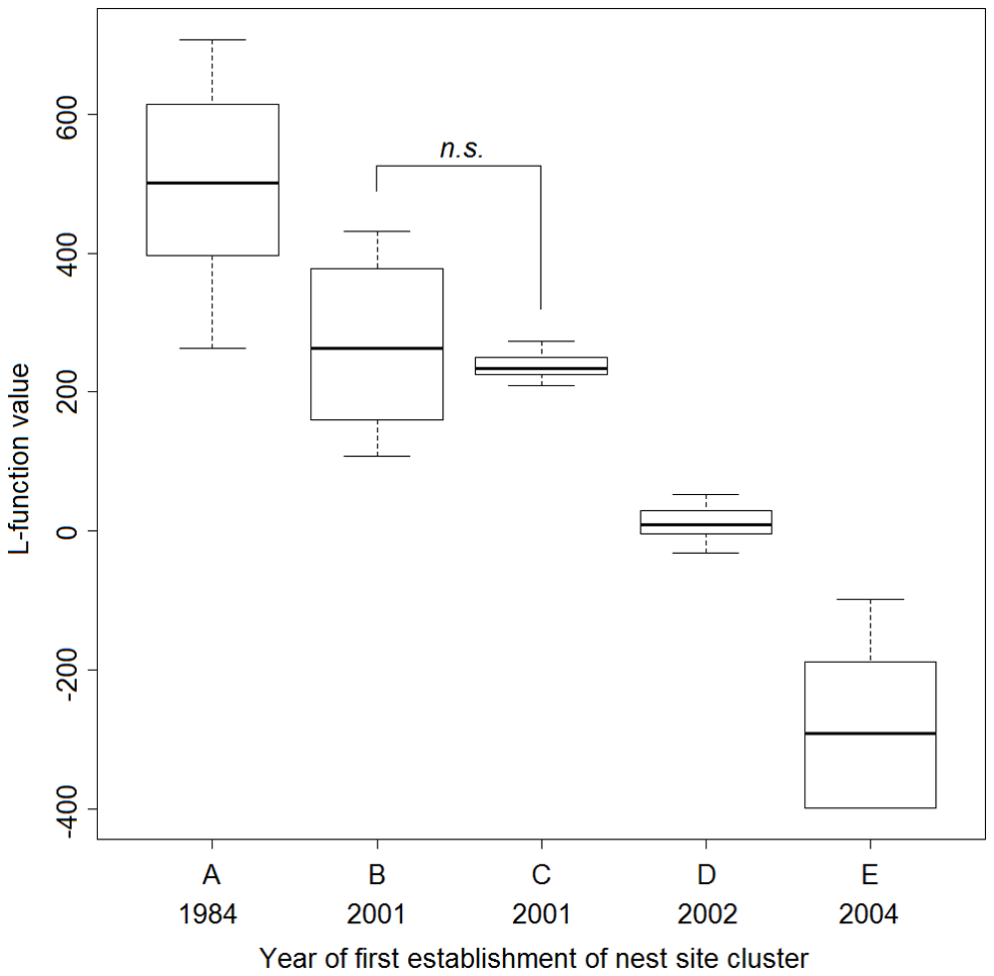
Variations in the spatial association between nest sites and winter-flooded rice fields over the years. Observed L-function values from bivariate L-function analysis for the five nest site clusters (A–E) established over the years are shown by box plots. The only pair with no significant difference is annotated based on post-hoc pairwise Tukey's HSD test (*P*≥0.05).

The bivariate L-function analyses showed that the spatial association between the nest sites and winter-flooded rice fields varied for the five nest site clusters (Figure 8). Cluster A showed a significant positive dependence on winter-flooded rice fields at distances of 500 m to 1,500 m. The positive dependence for Cluster B was marginally significant up to about 2,000 m, but significant at larger distances. For Cluster C, the spatial aggregation between nest sites and winter-flooded rice fields appeared to be significant at distances of 500 m to 1,000 m. In contrast, the bivariate L-function analysis failed to detect any significant spatial associations in Cluster D at all distances from 500 m to 3,000 m (Figure 8D). The nest sites in Cluster E are spatially independent of winter-flooded rice fields up to 1,500 m, although a segregation was detected at distances of 1,500 m to 2,500 m (Figure 8E). The result of one-way ANOVA confirmed the significance of differences in these spatial associations among the five nest site clusters (*F* = 45.62, *P*<0.001). The post-hoc test found the difference between each pair of means was significant at an alpha level of 0.05, except for the pair of Clusters B and C, which were established in the same year (2001) (Figure 9).

## Discussion

Our study shows that the nest sites of Crested Ibis and winter-flooded rice fields exhibit a significantly clumped distribution pattern. The nest sites are positively associated with the winter-flooded rice fields over the birds' entire distribution range. This association suggests that the clumping of the nest sites may be a response to the distribution of the winter-flooded rice fields (which can be patchy). Our results agree with the findings that birds' nests are spatially clumped in response to the heterogeneity in their available resources [43]–[45]. Other studies in the small relict Crested Ibis population (NW portion of Cluster A) focused on sample plots and showed that the winter-flooded rice fields had a significant effect on where the birds chose to nest [16]–[18]. Yu et al. [46] described how all the natal home ranges were close to winter-flooded rice fields with regard to the postfledging and natal dispersal of the Crested Ibis. Our findings are based on a spatial analysis of the entire population in 2009 and confirm the positive association between winter-flooded rice fields and nest sites during the recent expansion of the Crested Ibis's range.

We observed and confirmed variations in the spatial associations between the nest sites and winter-flooded rice fields among the nest site clusters (Figures 8 and 9). A spatial attraction (i.e. positive association) was found in Clusters A, B and C. The larger area of winter-flooded rice fields in Clusters B and C may increase the birds' food searching efficiency and reduce their energy consumption, which could, in turn, increase their reproductive fitness [47], [48]. Spatial dependencies were seen at different distances in Clusters A–C, suggesting that the distribution of winter-flooded rice fields is not the only habitat factor that has an effect on where the Crested Ibis choose to nest. The presence of suitable nesting trees, as well as human disturbances (e.g. hunting and mining), also limits the distribution of the breeding Crested Ibis [16]–[18], [22]. We noted that Cluster B showed a stronger dependence on the winter-flooded rice fields and was at a greater distance than Clusters A and C (Figure 8A–C). A plausible explanation seems to be that the larger patches of rice fields indicate more intensive human activities at lower elevations in Cluster B, where several strips of winter-flooded rice fields are near busy roads (observed during the fieldwork). The relatively high disturbance from the roads may constrain the establishment of nesting Crested Ibis compared to Cluster C, where there were more forest areas at higher elevations. However, Cluster A had a smaller extent of winter-flooded rice fields, but there were dozens of nest sites established here and they formed the largest cluster (Figure 6). Besides the habitat factor, the Crested Ibis' social behaviour, e.g. conspecifics attraction, may also be responsible for its pattern of nest site selection [49], [50]. However, we would expect fewer new nest sites to be established in this area in the future, since the specific foraging resources are limited and the birds' dependence on these resources is strong.

The preference of the Crested Ibis for winter-flooded rice fields is, however, no longer apparent after their expansion out from the relict population. Nest sites have been found to the south of the Han River (Cluster D) and on the floodplain (Cluster E) since 2002 and 2004, respectively, where the extent of winter-flooded rice fields is low to scarce. These two most recently established clusters are at a low elevation, and the Crested Ibis has shown no preference for nesting near winter-flooded rice fields (Figure 8D–E). Wetlands at low elevations, such as riverbanks, reservoirs and rice field channels, can act as alternative winter habitats and also provide the Crested Ibis with abundant food [51]–[53]. Despite the different dependencies seen for winter-flooded rice fields in their selection of nest sites, no significant variation in the number of successful nests and their breeding success at different altitudes was found [47]. Wang and Li [53] pointed out that the fledging success of the Crested Ibis did not differ between low- and high-elevation habitats. They also indicated that the low-elevation habitats, with a limited extent of winter-flooded rice fields, were capable of sustaining the Crested Ibis population inhabiting the area by offering the birds alternative foraging habitats. From a conservation point of view, the breeding success in relation to future habitat shifts should be investigated.

Intensive human disturbance (e.g. changing farming practices) and the high risk of human-induced mortality (due to hunting and use of toxic pesticides) historically led to a reduction in the Crested Ibis population at low elevations [20] and displaced them to more remote and higher montane areas where there was a lower human impact and consequently less risk [14], [53], [54]. The optimal habitats for survival and reproduction may remain unoccupied or under-utilised locally, depending on the proximity of alternative habitats with less disturbance [55]. Habitat use and selection change may indicate changing population constraints, such as less hunting pressure or less exposure to toxic pesticides in the floodplain area due to decades of conservation efforts. The rapid increase in the Crested Ibis population, with the observed shift in nesting habitats, suggests that these birds might have previously been restricted to suboptimal mountainous habitats at high elevations in the past [53]. The recently expanding population could be showing a return to more varied habitats. Furthermore, the changing role of winter-flooded rice fields in choosing where to nest may also suggest the Crested Ibis can show behavioural plasticity and an adaptive response to a heterogeneous environment. The behavioural plasticity in habitat selection and an ability to adapt to habitat heterogeneity may allow the birds to balance limitations experienced in the available habitats [56], [57]. Other waders, such as Terek Sandpipers *Xenus cinereus*, of which nests have been found in a wide range of habitats, also show behavioural plasticity in adapting to different environmental conditions, including urban areas [58]. Another example is the range expansion and adaptation to increased human activity reported for the Black Stork *Ciconia nigra* [56]. Similarly, the recovering Spanish Imperial Eagle *Aquila adalberti*, population has returned from its less populated habitats to the human-occupied plains it formerly favoured, now that it is legally protected and its habitats are sympathetically managed [3].

### Conservation implications

Our findings have important implications for the management and recovery planning of endangered species in general, and for Crested Ibis conservation, in particular. Firstly, natural wetlands have been decreasing globally and waterbirds have become increasingly dependent on rice agriculture for their survival [59]. Traditional rice production provided essential habitats for the survival and reproduction of Crested Ibis during the early stage of the relict population recovery, and still has a positive influence on their conservation. It is advocated that some patches of winter-flooded rice fields should be uniformly maintained especially in the area with less natural and semi-natural wetland, otherwise the Crested Ibis will find it difficult to survive. The conflicting interests of farmers and the Crested Ibis in these areas still need to be resolved by financial compensation.

Secondly, our study provides a better understanding of the contribution that traditional rice production can make to the re-introduction of the Crested Ibis. Although the population of Crested Ibis is now increasing in size and expanding its range, its current distribution range is still too limited for it to be confronted with the risks of natural catastrophes, such as disease. Efforts to reduce their risk of extinction have been carefully prioritised for potential re-introductions [20], [47]. We show that the dependence of the Crested Ibis on this specific habitat varies over space and that it has decreased over the years with the growth and expansion of the wild population. Our finding provides some guidance on the selection of re-introduction sites and indicates that the absence of winter-flooded rice fields is not a constraint to the recovery and expansion of the population. The conservation effort of widely encouraging rice cultivation practices that involve single cropping and extended periods of fallow flooding [13] may thus only be partially effective. We therefore recommend that an effort should be made to actively protect the existing winter-flooded rice fields, and to restore the functionality of natural and semi-natural wetlands for both the *in-situ* conservation and re-introduction of the Crested Ibis. Apart from the Crested Ibis, the knowledge of the historical habitat selection factors of many endangered species is limited. Our study supports the view that caution should be exercised when interpreting the habitat requirements of species whose distribution is restricted to just one or a few localities, particularly when that interpretation is based only on current habitats [3], [53].

Thirdly, we show that the investigation and monitoring of wildlife habitats using innovative remote sensing and GIS techniques can increase understanding of species-habitat relationships and can provide timely and accurate information for conservation and management decisions. This is particularly true when it comes to large areas with difficult access. In this study, we have demonstrated satellite images are effective for mapping winter-flooded rice fields across the study area. These spatially continuous, streams of habitat data are not, however, likely to be collected by ground surveys alone. Distinguishing man-made habitats from natural and semi-natural habitats may be challenging but it is very important, as the implications for management will vary greatly depending on the types of habitats and how they are created or maintained.

## Supporting Information

Figure S1 **Three levels of the extent of winter-flooded rice fields.** The area of winter-flooded rice fields within 3-km buffers around the nest sites was categorized into high-, medium- and low levels using Ward's hierarchical clustering method.
(DOC)

Table S1 **Probability rules used by the Bayesian expert system for land cover/land use mapping.**
(DOC)

Table S2 **Classification accuracy produced by the support vector machine (SVM) classifier and the integrated SVM and GIS expert system (hybrid classifier).**
(DOC)
