# Supplementary material for: Reduced Dependence of Crested Ibis on Winter-Flooded Rice Fields: Implications for Their Conservation
Source: PLoS One. 2014 May 29;9(5):e98690. doi: 10.1371/journal.pone.0098690 (PMC4038617; doi:10.1371/journal.pone.0098690)

**Figure S1.** **Three levels of the extent of winter-flooded rice fields.** The area of winter-flooded rice fields within 3-km buffers around the nest sites was categorized into high-, medium- and low levels using Ward’s hierarchical clustering method.


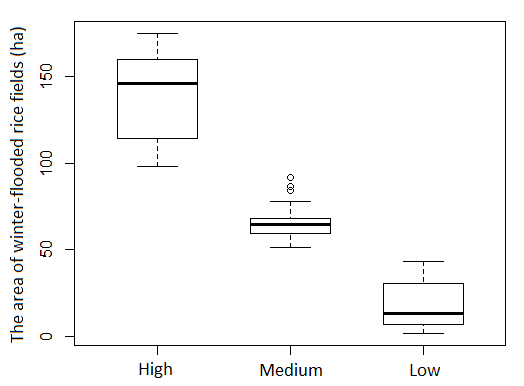

Supplement: Figure S1 — Three levels of the extent of winter-flooded rice fields. The area of winter-flooded rice fields within 3-km buffers around the nest sites was categorized into high-, medium- and low levels using Ward's hierarchical clustering method. (DOC) [file pone.0098690.s001.doc]
