# Supplementary material for: Reduced Dependence of Crested Ibis on Winter-Flooded Rice Fields: Implications for Their Conservation
Source: PLoS One. 2014 May 29;9(5):e98690. doi: 10.1371/journal.pone.0098690 (PMC4038617; doi:10.1371/journal.pone.0098690)
Supplement: Table S1 — Probability rules used by the Bayesian expert system for land cover/land use mapping. (DOC) [file pone.0098690.s002.doc]

**Table S1. Probability rules used by the Bayesian expert system for land cover/land use mapping.**

| Item of evidence | | Winter-dry rice field | Winter-flooded rice field | Shrub/Grass | Open water | Rain-fed field | Forest | Others |
| --- | --- | --- | --- | --- | --- | --- | --- | --- |
| Elevation (m) | < 500 | 0.4 | 0.05 | 0.1 | 0.3 | 0.2 | 0.05 | 0.3 |
|  | 500-600 | 0.3 | 0.2 | 0.15 | 0.2 | 0.5 | 0.1 | 0.15 |
|  | 600-800 | 0.25 | 0.3 | 0.3 | 0.1 | 0.25 | 0.2 | 0.1 |
|  | 800-1000 | 0 | 0.05 | 0.05 | 0.1 | 0.02 | 0.15 | 0.05 |
|  | 1000-1300 | 0.08 | 0.08 | 0.02 | 0 | 0 | 0.3 | 0.05 |
|  | >1300 | 0 | 0 | 0.03 | 0 | 0 | 0.95 | 0 |
|  |  |  |  |  |  |  |  |  |
| Terrain position | Gully | 0.4 | 0.4 | 0 | 0.1 | 0 | 0.01 | 0.05 |
|  | Lower mid-slope | 0.3 | 0.2 | 0.1 | 0.1 | 0.1 | 0.25 | 0.1 |
|  | Mid-slope | 0.05 | 0.05 | 0.3 | 0.1 | 0.3 | 0.3 | 0.1 |
|  | Upper mid-slope | 0.02 | 0 | 0.3 | 0 | 0.3 | 0.25 | 0.1 |
|  | Ridge | 0 | 0 | 0.3 | 0 | 0.3 | 0.2 | 0.1 |

Note: Values of zero in the table were replaced by a very small value close to zero in our calculations.
