# Supplementary material for: Reduced Dependence of Crested Ibis on Winter-Flooded Rice Fields: Implications for Their Conservation
Source: PLoS One. 2014 May 29;9(5):e98690. doi: 10.1371/journal.pone.0098690 (PMC4038617; doi:10.1371/journal.pone.0098690)
Supplement: Table S2 — Classification accuracy produced by the support vector machine (SVM) classifier and the integrated SVM and GIS expert system (hybrid classifier). (DOC) [file pone.0098690.s003.doc]

**Table S2. Classification accuracy produced by the support vector machine (SVM) classifier and the integrated SVM and GIS expert system (hybrid classifier).**

|  | SVM classifier | | Hybrid classifier | |
| --- | --- | --- | --- | --- |
| Class | Prod. Acc.(%) | User Acc.(%) | Prod. Acc.(%) | User Acc.(%) |
| Winter-dry rice field | 80.0 | 81.6 | 92.0 | 90.2 |
| Winter-flooded rice field | 88.1 | 71.8 | 89.3 | 90.4 |
| Shrub/Grass | 70.0 | 77.8 | 77.5 | 96.9 |
| Open water | 86.0 | 100.0 | 98.0 | 100.0 |
| Rain-fed field | 76.0 | 82.6 | 92.0 | 85.2 |
| Forest | 75.0 | 83.3 | 91.7 | 84.6 |
| Others | 90.0 | 84.9 | 94.0 | 94.0 |
| Overall accuracy (%) | 81.5 | | 90.8 | |
| Kappa | 0.78 | | 0.89 | |
